# Retinal Microvascular and Neuronal Changes Are Also Present, Even If Differently, in Adolescents with Type 1 Diabetes without Clinical Diabetic Retinopathy

**DOI:** 10.3390/jcm11143982

**Published:** 2022-07-08

**Authors:** Elisabetta Pilotto, Tommaso Torresin, Francesca Leonardi, Joaquin Gutierrez De Rubalcava Doblas, Giulia Midena, Carlo Moretti, Edoardo Midena

**Affiliations:** 1 Department of Ophthalmology, University of Padova, 35128 Padova, Italy; tommaso.torresin@unipd.it (T.T.); edoardo.midena@unipd.it (E.M.); 2 Department of Ophthalmology, “De Gironcoli” Hospital, Conegliano, 31015 Treviso, Italy; leonardifrancesca88@gmail.com; 3 Pediatric Diabetes Unit, Department of Women’s and Children’s Health, University of Padova, 35128 Padova, Italy; joaquin.gutierrezderubalcava@aopd.veneto.it (J.G.D.R.D.); carlo.moretti@aopd.veneto.it (C.M.); 4 IRCCS, Fondazione Bietti, 00198 Rome, Italy; giulia.midena@fondazionebietti.it

**Keywords:** type 1 diabetes mellitus, adolescents, OCT, OCT angiography, retinal layers, glycemic indices, continuous glucose monitoring

## Abstract

The purpose of this study was to evaluate retinal changes in adolescents with childhood-onset, long-lasting type 1 diabetes mellitus (T1D). Patients and healthy controls (HC) underwent optical coherence tomography (OCT) and OCT-angiography (OCTA). Individual macular layers, peripapillary retinal nerve fiber layer (pRNFL), and vascular parameters (vessel area density (VAD), vessel length fraction (VLF) and vessel diameter index (VDI)) of macular superficial vascular (SVP), intermediate (ICP), deep (DCP) and radial peripapillary capillary plexuses (RPCP) were quantified. Thirty-nine patients (5 with (DR group) and 34 without (noDR group) diabetic retinopathy) and 20 HC were enrolled. The pRNFL and ganglion cell layer (GCL) were thicker in noDR compared to HC and DR, reaching statistically significant values versus HC for some sectors. At the macular level, VAD and VLF were reduced in DR versus HC in all plexuses, and versus noDR in SVP (*p* < 0.005 for all). At the RPCP level, VAD and VDI were increased in noDR versus HC, significantly for VDI (*p* = 0.0067). Glycemic indices correlated to retinal parameters. In conclusion, in T1D adolescents, retinal capillary and neuronal changes are present after long-lasting disease, even in the absence of clinical DR. These changes modify when clinical retinopathy develops. The precocious identification of specific OCT and OCTA changes may be a hallmark of subsequent overt retinopathy.

## 1. Introduction

Type 1 diabetes mellitus (T1D) is the most common metabolic disorder of childhood and adolescence, and its prevalence has been increasing over the last years [1]. Although pediatric subjects with T1D are at low risk of diabetic retinopathy (DR), a high prevalence of DR and proliferative DR has been reported after an 18-year follow-up in patients who had T1D since childhood [2]. Early (pre-clinical) identification of retinal changes in young patients with T1D may be of clinical value for monitoring retinal disease. With the advent of optical coherence tomography angiography (OCTA), early pre-clinical retinal vascular changes have been largely investigated in adults with DM, mainly with T2D [3,4,5,6,7], but less extensively in children and adolescents with T1D [8,9,10,11,12]. Diabetic retinopathy has been historically considered primarily a retinal microvascular disease. Currently, scientific data suggests that in diabetes, the retinal neurovascular unit, which encompasses endothelial cells and pericytes to retinal neurons and glial cells (microglia and macroglia), is the primary site of retinal damage [13,14]. The neurovascular unit impairment induces specific morphological changes of retinal layers, detectable by means of OCT, which seem to precede clinical signs of DR [4,15,16,17,18,19,20,21]. When these morphological and vascular retinal changes have been correlated to glycated hemoglobin (HbA1c), a classic metabolic parameter, inconsistent results have been reported [8]. The adoption of continuous glucose monitoring (CGM) in T1D management, as recently recommended by the Advanced Technologies and Treatments for Diabetes (ATTD) consensus, allows for obtaining more useful indices of patients’ glycemic control that can integrate the classic glycemic parameters [22].

The aim of this study was to evaluate retinal changes, using both OCT and OCTA, in a cohort of adolescents referred to a tertiary pediatric diabetes unit affected by long-lasting T1D and to correlate these findings with CGM metrics and HbA1c.

## 2. Materials and Methods

### 2.1. Participants

This was an observational cross-sectional study that consecutively enrolled all adolescents with childhood-onset and long-lasting (>10 years) T1D that were referred to the Pediatric Diabetes Unit of the Padova University Hospital. Informed consent was obtained from each subject’s legal guardian or enrolled subject (>18 years old). The research was carried out in accordance with the Declaration of Helsinki. Local ethics committee approval for the study was obtained. The inclusion criteria were: (i) age ≤ 20 years old; (ii) diagnosis of T1D according to the World Health Organization classification [23]; (iii) duration of the disease >10 years. Exclusion criteria were: (i) neurodegenerative diseases or other neurological disorders independent from diabetes; (ii) history of prematurity; (iii) any alteration of the retina or choroid that could modify OCT analysis (i.e., hereditary retinal dystrophy, vitreoretinal diseases, myopia or hyperopia more than 3 diopters and history of uveitis); (iv) history of glaucoma or intraocular pressure ≥ 21 mmHg. A control group, composed of healthy subjects, underwent the same examinations. All enrolled subjects underwent a complete ophthalmological examination, including refraction and best-corrected distance visual acuity (BCVA) measurement, anterior segment examination, ophthalmoscopy and fundus biomicroscopy. They also underwent spectral-domain OCT and OCTA examination after pupillary dilation with 1% tropicamide. 

### 2.2. Imaging

OCT and OCTA were performed using the Spectralis HRA + OCT2 platform (Heidelberg Engineering, Heidelberg, Germany), as previously described [24,25,26]. The following scans were captured: (i) a 20° × 20° volumetric macular map centered on the foveola for the measurements of thickness and volume of macular retinal layers; (ii) a circumpapillary ring scan with a diameter of 3.5 mm centered on the optic nerve head with a resolution of 100 ART for the measurement of the peripapillary retinal nerve fiber layer (pRNFL) thickness; (iii) a 10° × 10° OCTA map centered on the foveola for identification of the macular vascular plexuses; (iv) a 10° × 10° OCTA map centered on the optic nerve head for detection of the radial peripapillary capillary plexus (RPCP).

An ETDRS (Early Treatment for Diabetic Retinopathy Study) grid centered onto the fovea subdivided the macular area into nine parts according to the incorporated Spectralis software, consisting of a central circular zone with 1 mm diameter and inner and outer rings of 3 and 6 mm diameter, respectively. The internal and external rings were subdivided into four quadrants. The automatic segmentation software of the OCT device (Heidelberg Eye Explorer; Heidelberg Engineering) was used to segment the retinal layers. The algorithm detected 11 separation markers. For this study, the following retinal layers were considered: retinal nerve fiber layer (RNFL), ganglion cell layer (GCL), inner plexiform layer (IPL); inner nuclear layer (INL); outer plexiform layer (OPL); outer nuclear layer (ONL); outer retinal layer (ORL: external limiting membrane plus myoid zone, ellipsoid zone, and outer segments of the photoreceptors plus cone interdigitation with RPE and RPE/Bruch’s membrane complex) as previously described [24]. After automated segmentation, manual refinement was eventually performed in case of errors or artifacts. The mean volume of the individual retinal layers was automatically provided by the software. Moreover, the mean thickness of the nine ETDRS subfields for each individual layer was considered for the study [24].

As regards OCTA, the automatic segmentation algorithm (Heyex Software) identified the superficial vascular plexus (SVP) from RNFL to 17 μm above IPL (IPL-), the intermediate capillary plexus (ICP) from IPL- to 22 μm below IPL (IPL+), the deep capillary plexus (DCP) from IPL+ to OPL in the macular area, and RPCP in the optic disc area [27]. Quantitative analysis of the vascular plexuses in the OCTA en-face images was performed using the open-source, available ImageJ software (National Institutes of Health, Bethesda, MD, USA). Three parameters were analyzed: vessel area density (VAD), vessel length fraction (VLF) and vessel diameter index (VDI). To obtain high-quality OCTA images, a signal strength (SS) of more than 30 in “Q score” (on a scale of 0 to 40 for Spectralis, Heidelberg, Germany) was required during OCTA acquisition [27].

A skilled operator performed all OCT and OCTA scans. All examinations were performed in the morning to avoid possible diurnal variation. A masked operator performed all OCT and OCTA measurements.

### 2.3. Systemic Glycemic Indices

The following standardized CGM metrics were considered: mean glucose (mg/dL), glycemic variability (standard deviation value of blood glucose: SD), “time in range” (TIR, % of readings within 70–180 mg/dL) and “time below range” (TBR, % of readings < 70 mg/dL). Moreover, HbA1c (%) and advanced glycation end-products (AGE, AU) were quantified. For each of these parameters, the average and range of variability (difference between the minimum and maximum) were obtained considering the three-monthly values of the last year. 

### 2.4. Statistical Analysis

The computation of the parameters was made by calculating the usual indicators provided by the descriptive statistics: arithmetic mean, standard deviation and range of variability (maximum value–minimum value) for the quantitative parameters and absolute and relative frequency distribution (percentage) for qualitative ones (gender and presence or absence of DR). The distribution of the data was tested for normality. The inferential analysis saw the comparison of eyes with diabetic retinopathy and those without diabetic retinopathy with respect to controls in relation to OCT macular parameters, pRNFL and OCTA indices of macula and papilla. For this analysis, a mixed-effects analysis of variance (PROC MIXED) model was used with repeated measurements in both patients’ eyes and, in the case of retinal thicknesses, in the 9 ETDRS sectors. Specific comparisons were also made at the single sector level. A further analysis was conducted on diabetic patients only to evaluate the correlation between retinal findings (OCT and OCTA data) and systemic glycemic indices and disease duration. A multiple linear regression model (adjusted for replications of measurements considering both eyes of each patient) was estimated for each variable. A test of statistical significance of the regression coefficient was made. The regression coefficient was used to express the magnitude of the correlation and its direction (direct if the sign of the coefficient was positive, inverse if it was negative).

All analyses were carried out using SAS 9.4 statistical software (SAS Institute, Cary, NC, USA) on a personal computer. The results of the statistical tests were interpreted as significant if *p* < 0.05.

## 3. Results

### 3.1. Population

A total of 51 T1D patients and 20 controls were enrolled. Overall, 12 patients were excluded from the study: 2 (4%) due to poor compliance and 10 (19.6%) due to myopic refractive defect (>3 diopters). Therefore, 39 patients (78 eyes) and 20 healthy controls (40 eyes, HC group) were included in the analysis. Additionally, 34 T1D patients had no clinical sign of DR (noDR group, 68/78 eyes, 87.2%), and 5 had mild DR according to the Clinical Diabetic Retinopathy Disease Severity Scale (DR group, 10/78 eyes, 12.8%). The mean duration of diabetes was 12.5 ± 2.1 years in noDR and 12.8 ± 2.0 years in DR (*p* = 0.8202). The two diabetic groups did not differ for any of the analyzed systemic glycemic indices. The mean age of noDR patients (19 females (55.88%) and 15 males (44.12%)) was 17.2 ± 2.0 years. The mean age of DR patients (3 females (60%) and 2 males (40%)) was 17.0 ± 1.6 years. The mean age of controls (12 females (60%) and 8 males (40%)) was 17.3 ± 3.1 years. There was no significant difference in gender and age distribution among groups (*p* > 0.05 for all). Diabetic population demographic and disease characteristics are reported in Table 1. BCVA was 85 ± 0 ETDRS letters in all groups.

### 3.2. Retinal Layers Volume and Thickness

GCL volume was always higher in noDR, significantly so when compared to HC (1.19 ± 0.08 mm^3^ vs. 1.14 ± 0.08 mm^3^, *p* = 0.0433). ORL volume significantly increased in noDR and in DR versus HC (*p* = 0.0346 and *p* = 0.0340, respectively), but not between noDR and DR (*p* = 0.2872). All volume data are fully reported in Table 2.

**Figure 1:**
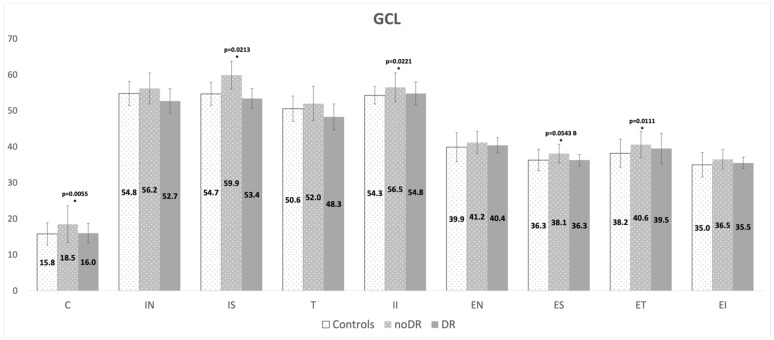
Ganglion cell layer (GCL) mean thickness (mm) comparison between controls, diabetic eyes without clinical signs of diabetic retinopathy (noDR) and diabetic eyes with clinical signs of diabetic retinopathy (DR) in the nine sectors from the ETDRS grid: C: central, IN: internal nasal, IS: internal superior, IT: internal temporal, II: internal inferior, EN: external nasal, ES: external superior, ET: external temporal, EI: external inferior. * significant difference compared to controls. B: borderline significance.

Full retina thickness was always higher in noDR compared to HC and DR, reaching statistical significance versus HC just for some sectors. No significant differences were present between DR and HC (Table S1). When single retinal layers were considered, the main differences concerned pRNFL, GCL and ORL layers. pRNFL and GCL were always thicker in noDR compared to HC and DR, being statistically significant versus HC just for some sectors (Figure 1 and Table 3). No significant differences in GCL and pRNFL thickness were present between DR and HC. ORL was always thicker in noDR and DR versus HC, reaching statistical significance for some sectors. No significant differences were present in ORL thickness between noDR and DR, except for a single sector in which this layer was thicker in DR versus noDR (Table 4). 

The remaining retinal layer thickness data and comparisons between groups are fully reported in Supplementary Materials (Table S1).

### 3.3. OCT Angiography Parameters

**Figure 2:**
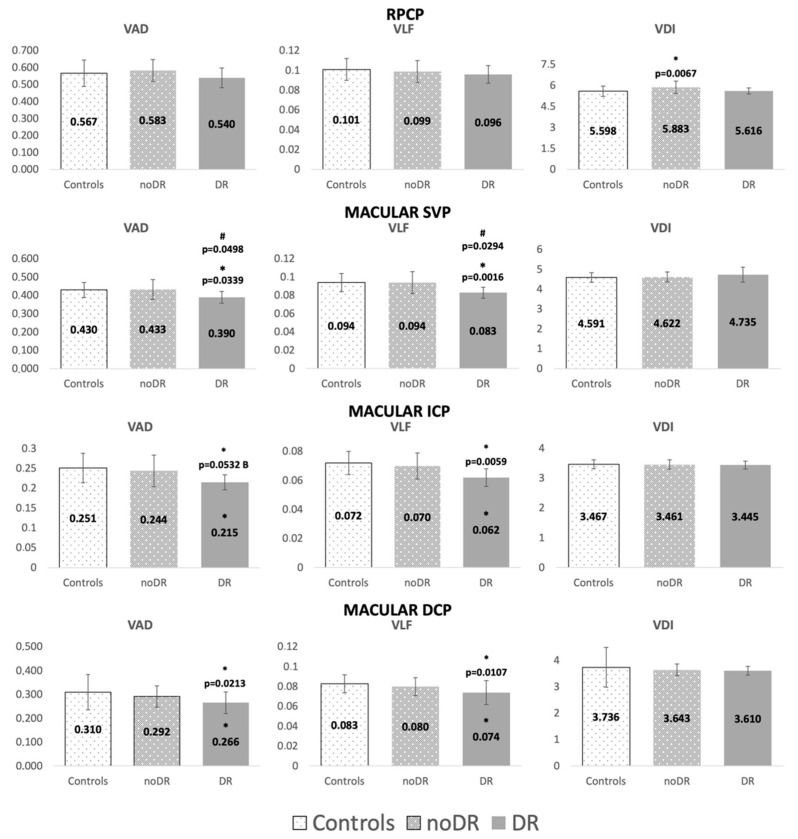
Representation of vessel area density (VAD), vessel length fraction (VLF), and vessel diameter index (VDI) of the radial peripapillary capillary plexus (RPCP), superficial vascular plexus (SVP), intermediate (ICP) and deep capillary plexus (DCP). Comparison between controls, diabetic eyes without clinical signs of diabetic retinopathy (noDR) and diabetic eyes with clinical signs of diabetic retinopathy (DR). * Statistically significant difference between DR and controls; # statistically significant difference between DR and noDR group. B: borderline significance.

At the macular plexuses level, VAD and VLF were reduced in DR, significantly when compared to HC in all plexuses, and just in the SVP when compared to noDR. They did not differ between noDR and HC. VDI of all macular plexuses did not differ among groups. At the RPCP level, VAD and VDI were always increased in noDR compared to DR and HC, reaching statistical significance just for VDI versus HC. VLF of RPCP did not differ among groups (Figure 2).

### 3.4. Correlations of Glycemic Indices with OCT and OCTA Parameters

Significant correlations of glycemic indices with total retinal and individual retinal layer volume and OCTA parameters are fully reported in Table 5.

Many glycemic indices correlated to OCTA vascular parameters of the ICP: mean glucose, HbA1c mean, and SD mean were inversely correlated, while TIR mean was directly correlated to all OCTA parameters (VAD, VLF and VDI). HbA1c variability was inversely correlated to the total retina, GCL, IPL and ONL volume. 

Correlations among individual retinal layers thickness of the ETDRS sectors and glycemic indices were also calculated. Across all searched correlations, the statistical significance was mostly sporadic with single ETDRS sectors; therefore, they have not been reported.

## 4. Discussion

In T1D adolescents with long-lasting disease, retinal capillary networks changed, but differently according to the presence or absence of clinical DR. If in patients with clinical DR a diffuse decrease in vascular vessel density was detectable, involving all macular plexuses, in patients with noDR, a mild, not significant reduction of OCTA parameters was present and limited to the deeper plexuses (ICP and DCP). Golębiewska et al. detected no difference in vessel density between T1D patients with no signs of DR and healthy controls after a mean disease duration of 6 years [28]. Our results confirmed that even after a longer disease duration (>10 years), macular plexuses were not significantly affected in patients with no clinical DR. Mameli et al. reported a decrease in macular vascular density in young T1D compared to controls [8]. However, our results cannot be compared to those of Mameli et al. for different reasons. First, they did not separately analyze patients without and with DR, which was present in a small percentage of their cases. Second, they divided the retinal capillaries into superficial and deep capillary plexuses, with the anatomic ICP incorporated into or split between the other two plexuses [8]. Retinal capillary beds are functionally independent neurovascular units [29], and in eyes with clinical signs of DR, when ICP is separately evaluated, parallel changes of ICP and DCP have been detected [30,31,32]. Therefore, OCTA studies adopting segmentation protocols that include ICP within SVP may provide different results [30,33,34]. 

In the RPCP, blood flow was increased in noDR compared to HC, mainly due to vascular dilation (VDI). The RPCP is a distinct retinal vascular network located in the peripapillary retinal nerve fiber layer, characterized by a specific radial distribution of capillaries, tightly coupling and supplying nerve fibers [35]. At this level, retinal blood flow is highly correlated to neural activity in a strict neurovascular metabolic coupling [36]. We detected thickening of pRNFL in noDR. A higher flow at this level may be secondary to an autoregulatory response to increased metabolic demand, corroborating what was previously hypothesized [15]. In DR eyes, vascular dilation was not detectable, as VDI reached HC values, and VAD was lower. Vascular dilation in the RPCP could therefore be an early marker of subsequent clinical diabetic microvascular changes in young T1D patients. Further follow-up OCTA studies should include the RPCP in their evaluation.

It has been demonstrated that retinal neurodegeneration occurs in diabetic patients, even in the absence of clinical signs of DR [3,34,37]. In adolescents with noDR, we detected an increase in GCL volume compared to HC, while there were no differences between DR and HC. Our results cannot be compared with those reported in previous studies, in which the ganglion cell complex, which includes the GCL and IPL, has been measured in young diabetic patients [31,32] pRNFL thickness was also higher in noDR, while it did not differ between DR and HC. Differently from our results, El Fayoumi et al. detected a significant reduction in pRNFL thickness in young T1D and no DR [31]. However, these authors included patients with a myopic refractive defect (up to 8D), which is accompanied by the thinning of retinal layers [38]. Thickening of GCL and RNFL has also been previously reported in adults with T1D and no DR [7]. In immunohistochemical studies, ganglion cells responded to hyperglycemia with an increase in the volume of their body, thickening of the axons and a greater number of dendritic branches [39]. Reactive gliosis and subsequent neural apoptosis are both histological features of retinal neurodegeneration in DM [15]. Our clinical results suggest that reactive gliosis, with thickening of GCL and pRNFL, precedes neural apoptosis, with progressive thinning of these retinal layers when clinical signs of DR develop [40]. Follow-up studies of young T1D patients might corroborate this hypothesis. 

In the present study, the outer retina (ORL) was increased in volume and thickness in both noDR and DR patients. To date, outer retina changes have never been analyzed in young patients with T1D. Different elements and cells contribute to the outer retina, including the photoreceptors, retinal pigment epithelium cells and Bruch’s membrane. Therefore, an increase in outer retina volume and/or thickness may have a different origin [35,36,41]. Given the anatomical and functional complexity of the outer retina, it would be useful in the future to separately analyze these individual layers, discriminating which of them is primarily affected in young patients with diabetes.

Correlations between glycemic indices and OCTA vascular changes in diabetic patients have been largely debated in recent years. Wysocka-Mincewicz et al., in an OCTA study, reported an inverse correlation between vessel density of the SVP and HbA1c [42]. This study was very heterogeneous in terms of diabetes duration and did not include patients with signs of DR. Other OCTA studies reported no correlation between HbA1c and macular retinal vessel density, suggesting that “time in range” (TIR) monitoring could be more promising [8,43,44]. In the present study, mean TIR positively correlated to ICP indexes. Beyond HbA1c and TIR, we analyzed additional glycemic indices, many of which correlated with ICP or SVP vascular parameters. The results of the present study underline that retinal vascular impairment is correlated to glycemic metabolic control, and macular plexuses are differently influenced by glycemic control, with superficial plexus (SVP) more sensitive to glycemic variability (HbA1c variability) and deep plexuses (mainly ICP) to poor chronic glycemic control (mean TIR, mean glucose, mean H1A1c, SD) as revealed by the numerous correlations with the analyzed glycemic indices. 

The main limitation of our study was the small sample size. However, the strength of the study was the inclusion of a pediatric cohort that was homogeneous both in terms of age and duration of diabetes and treatment regimen, coming from a single tertiary pediatric diabetes unit. A longitudinal study in a larger cohort of pediatric patients could be useful to strengthen our findings and allow us to detect imaging biomarkers of disease progression reflecting microvascular complications and identify high-risk patients”.

## 5. Conclusions

In conclusion, in this study, we detected that in adolescents with childhood-onset T1D, morphologic and microvascular retinal changes are present after long-lasting disease, even if clinical signs of DR are absent. However, these changes differ when clinical retinopathy develops and differently involve single plexuses and layers. In patients without clinical DR, vascular flow is increased in RPCP, with thickening of pRNFL and GCL, suggesting an increased metabolic demand. When clinical signs of DR are present, macular flow is compromised in all macular plexuses. The precocious identification of specific OCT and OCTA changes may be a hallmark of subsequent overt retinopathy. Moreover, variability in glycemic indices more strongly correlates to retinal structural and vascular changes than the sole HbA1c value, underling the importance of considering different glycemic indices rather than HbA1c alone. 

## Figures and Tables

**Table 1:** Demographic and disease characteristics.

	HC Group	noDR Group	DR Group	*p*-Value noDR vs. DR
Eyes	40	68	10	
Mean age ± SD (years)	17.3 ± 3.1	17.2 ± 2.0	17.0 ± 1.6	0.8602 ^a^
Mean duration T1D ± SD (years)	n.a.	12.5 ± 2.1	12.8 ± 2.0	0.8202 ^a^
Mean glucose ± SD (mg/dL)	n.a.	178.2 ± 30.9	179.6 ± 14.0	0.8640 ^a^
Glycemic variability (SD) ± SD (mg/dL)	n.a.	80.0 ± 14.7	84.4 ± 7.0	0.3392 ^a^
TIR ± SD (%)	n.a.	46.6 ± 11.2	48.3 ± 3.9	0.5495 ^a^
TBR ± SD (%)	n.a.	9.7 ± 6.9	9.3 ± 4.7	0.8806 ^a^
HbA1c ± SD (%)	n.a.	7.6 ± 1.0	7.7 ± 0.9	0.7737 ^a^
AGE ± SD (AU)	n.a.	1.3 ± 0.2	1.5 ± 0.4	0.2478 ^a^

noDR: eyes without diabetic retinopathy; DR: eyes with diabetic retinopathy; SD: standard deviation; T1D: type 1 diabetes; TIR: time in range; TBR: time below range; HbA1c: glycated haemoglobin; AGE: advanced glycation end-products. ^a^ Student *t*-test for independent samples. n.a.: not applicable.

**Table 2:** Full retina and individual macular retinal layers volume.

	HC Group Mean ± SD (mm^3^)	noDR Group Mean ± SD (mm^3^)	DR Group Mean ± SD (mm^3^)	*p*-Value noDR vs. HC	*p*-Value DR vs. HC	*p*-Value DR vs. noDR
Full retina	8.79 ± 0.33	8.98 ± 0.35	8.77 ± 0.29	0.0632	0.8716	0.8892
RNFL	0.90 ± 0.06	0.94 ± 0.09	0.88 ± 0.06	0.1460	0.2266	0.4465
GCL	1.14 ± 0.08	1.19 ± 0.08	1.15 ± 0.05	**0.0433**	0.9625	0.7731
IPL	0.94 ± 0.07	0.97 ± 0.06	0.94 ± 0.04	0.1409	0.7403	0.8478
INL	0.99 ± 0.07	1.00 ± 0.06	0.97 ± 0.04	0.6313	0.5663	0.5393
OPL	0.80 ± 0.07	0.79 ± 0.05	0.80 ± 0.05	0.7010	0.9923	0.7368
ONL	1.77 ± 0.17	1.80 ± 0.17	1.73 ± 0.18	0.5484	0.7663	0.9041
ORL	2.26 ± 0.06	2.29 ± 0.07	2.32 ± 0.06	**0.0346**	**0.0340**	0.2872

HC: healthy controls; noDR: eyes without diabetic retinopathy; DR: eyes with diabetic retinopathy; SD: standard deviation; RNFL: retinal nerve fiber layer; GCL: ganglion cells layer; IPL: inner plexiform layer; INL: inner nuclear layer; OPL: outer plexiform layer; ONL: outer nuclear layer; ORL: outer retinal layer. Significant *p*-values in bold.

**Table 3:** Peripapillary retinal nerve fiber layer thickness.

Sector	HC Group Mean ± SD (mm)	noDR Group mean ± SD (mm)	DR Group Mean ± SD (mm)	*p*-Value noDR vs. HC	*p*-Value DR vs. HC	*p*-Value DR vs. noDR
Global	101.0 ± 9.6	106.3 ± 9.6	103.2 ± 9.5	0.2025	0.6723	0.8380
Nasal	75.2 ± 16.9	78.3 ± 13.5	74.1 ± 10.4	0.4709	0.9533	0.9935
Nasal Superior	115.1 ± 22.8	116.7±19.2	106.8 ± 17.1	0.7175	0.2667	0.2943
Temporal Superior	147.3 ± 15.0	148.6 ± 18.3	149.6 ± 17.1	0.7778	0.6617	0.3378
Temporal	72.7 ± 16.3	77.2 ± 16.8	73.1 ± 8.4	0.2860	0.8755	0.9826
Temporal Inferior	147.4 ± 13.8	155.7 ± 17.0	156.1 ± 14.0	**0.0447**	0.1715	0.3952
Nasal Inferior	106.9 ± 26.7	121.5 ± 23.3	118.1 ± 26.4	**0.0004**	0.0853	0.8826

HC: healthy controls; noDR: eyes without diabetic retinopathy; DR: eyes with diabetic retinopathy; SD: standard deviation. Significant *p*-values in bold.

**Table 4:** Macular outer retinal layer thickness.

Sector	HC Group Mean ± SD (mm)	noDR Group Mean ± SD (mm)	DR Group Mean ± SD (mm)	*p*-Value noDR vs. HC	*p*-Value DR vs. HC	*p*-Value DR vs. noDR
Central	93.2 ± 4.5	94.3 ± 3.5	93.6 ± 4.7	0.1515	0.9135	0.2725
Internal Nasal	84.1 ± 2.9	85.3 ± 2.7	85.3 ± 2.4	0.0957	0.4109	0.8446
Internal Superior	81.4 ± 2.6	83.1 ± 2.6	83.4 ± 2.4	**0.0209**	0.1472	0.8472
Internal Temporal	82.4 ± 3.5	83.9 ± 2.9	84.1 ± 2.8	**0.0378**	0.2225	0.9621
Internal Inferior	80.8 ± 2.9	82.2 ± 2.7	82.8 ± 2.8	**0.0556 B**	0.1472	0.5700
External Nasal	79.9 ± 2.0	81.1 ± 2.6	82.6 ± 2.4	0.0944	**0.0363**	0.0760
External Superior	79.1 ± 2.4	80.8 ± 2.7	82.0 ± 2.5	**0.0253**	**0.0281**	0.1516
External Temporal	77.8 ± 2.8	78.9 ± 2.8	81.1 ± 2.9	0.1038	**0.0097**	**0.0123**
External Inferior	77.2 ± 2.3	79.0 ± 2.8	80.6 ± 2.9	**0.0129**	**0.0091**	0.0676

HC: healthy controls; noDR: eyes without diabetic retinopathy; DR: eyes with diabetic retinopathy; SD: standard deviation. Significant *p*-values in bold. B: borderline value.

**Table 5:** Correlation between glycemic indices and OCT and OCTA parameters.

	OCT (Volume)	Correlation: (+ or −) *p* = Value	OCTA (Vascular Parameter)	Correlation: (+ or −) *p* = Value
Mean glucose	none		ICP (VAD, VLF, VDI)	(−) *p* = 0.0035, *p* = 0.0168, *p* = 0.0226
Mean glucose variability	ORL	(−) *p* = 0.0402	none	
Time in range mean	none		ICP (VAD, VLF, VDI)	(+) *p* = 0.0095, *p* = 0.0470, *p* = 0.009
Time below range mean	none		RPCP (VAD, VLF)	(+) *p* = 0.0539, *p* = 0.0395
HbA1c mean	none		ICP (VAD, VLF, VDI)	(−) *p* = 0.0039; *p* = 0.0211; *p* = 0.0184
HbA1c variability	Total Retina	(−) *p* = 0.0312	SVP (VAD, VLF)	(−) *p* = 0.0010, *p* = 0.0047
	GCL	(−) *p* = 0.0311	DCP (VLF)	(+) *p* = 0.0320
	IPL	(−) *p* = 0.0241		
	ONL	(−) *p* = 0.0515		
Glycemic variability (SD) mean	none		ICP (VAD, VFL, VDI)	(−) *p* = 0.0030, *p* = 0.0354, p = 0.0045

GCL: ganglion cells layer; IPL: inner plexiform layer; INL: inner nuclear layer; ONL: outer nuclear layer; ORL: outer retinal layers; SCP: superficial vascular plexus; ICP: intermediate capillary plexus; DCP: deep capillary plexus. VAD: vessel area density; VLF: vessel length fraction; VDI: vessel diameter index. AGE: advanced glycation end-products. Type of correlation: −: negative correlation, +: positive correlation. Only significant correlations are reported.

## Data Availability

The data presented in this study are available in the article. Eventual additional data are available on request from the corresponding author.

## Supplementary Materials

The following supporting information can be downloaded at: https://www.mdpi.com/article/10.3390/jcm11143982/s1, Table S1: Full retina and single retinal layer thickness.

## References

[1] Lawrence J.M., Divers J., Isom S., Saydah S., Imperatore G., Pihoker C., Marcovina S.M., Mayer-Davis E.J., Hamman R.F., Dolan L. (2021). Trends in prevalence of type 1 and type 2 diabetes in children and adolescents in the US, 2001–2017. JAMA J. Am. Med. Assoc..

[2] Hautala N., Hannula V., Palosaari T., Ebeling T., Falck A. (2014). Prevalence of diabetic retinopathy in young adults with type 1 diabetes since childhood: The oulu cohort study of diabetic retinopathy. Acta Ophthalmol..

[3] Vujosevic S., Midena E. (2013). Retinal layers changes in human preclinical and early clinical diabetic retinopathy support early retinal neuronal and Müller cells alterations. J. Diabetes Res..

[4] van Dijk H.W., Verbraak F.D., Kok P.H.B., Garvin M.K., Sonka M., Lee K., Devries J.H., Michels R.P.J., van Velthoven M.E.J., Schlingemann R.O. (2010). Decreased retinal ganglion cell layer thickness in patients with type 1 diabetes. Investig. Ophthalmol. Vis. Sci..

[5] Srinivasan S., Pritchard N., Sampson G.P., Edwards K., Vagenas D., Russell A.W., Malik R.A., Efron N. (2016). Retinal tissue thickness in type 1 and type 2 diabetes. Clin. Exp. Optom..

[6] Scarinci F., Picconi F., Virgili G., Giorno P., Di Renzo A., Varano M., Frontoni S., Parravano M. (2017). Single retinal layer evaluation in patients with type 1 diabetes with no or early signs of diabetic retinopathy: The first hint of neurovascular crosstalk damage between neurons and capillaries?. Ophthalmologica.

[7] Araszkiewicz A., Zozulińska-Ziółkiewicz D., Meller M., Bernardczyk-Meller J., Piłaciński S., Rogowicz-Frontczak A., Naskrȩt D., Wierusz-Wysocka B. (2012). Neurodegeneration of the retina in type 1 diabetic patients. Pol. Arch. Med. Wewn..

[8] Mameli C., Invernizzi A., Bolchini A., Bedogni G., Giani E., MacEdoni M., Zuccotti G., Preziosa C., Pellegrini M. (2019). Analysis of retinal perfusion in children, adolescents, and young adults with type 1 diabetes using optical coherence tomography angiography. J. Diabetes Res..

[9] Li T., Jia Y., Wang S., Wang A., Gao L., Yang C., Zou H. (2019). Retinal microvascular abnormalities in children with type 1 diabetes mellitus without visual impairment or diabetic retinopathy. Investig. Ophthalmol. Vis. Sci..

[10] Niestrata-Ortiz M., Fichna P., Stankiewicz W., Stopa M. (2019). Enlargement of the foveal avascular zone detected by optical coherence tomography angiography in diabetic children without diabetic retinopathy. Graefes Arch. Clin. Exp. Ophthalmol..

[11] Dimitrova G., Chihara E., Takahashi H., Amano H., Okazaki K. (2017). Quantitative retinal optical coherence tomography angiography in patients with diabetes without diabetic retinopathy. Investig. Ophthalmol. Vis. Sci..

[12] Simonett J.M., Scarinci F., Picconi F., Giorno P., De Geronimo D., Di Renzo A., Varano M., Frontoni S., Parravano M. (2017). Early microvascular retinal changes in optical coherence tomography angiography in patients with type 1 diabetes mellitus. Acta Ophthalmol..

[13] Antonetti D.A., Klein R.E., Gardner T.W. (2012). Diabetic retinopathy. N. Engl. J. Med..

[14] Das A., McGuire P.G., Rangasamy S. (2015). Diabetic macular edema: Pathophysiology and novel therapeutic targets. Ophthalmology.

[15] Simó R., Hernández C. (2014). Neurodegeneration in the diabetic eye: New insights and therapeutic perspectives. Trends Endocrinol. Metab..

[16] De Clerck E.E.B., Schouten J.S.A.G., Berendschot T.T.J.M., Kessels A.G.H., Nuijts R.M.M.A., Beckers H.J.M., Schram M.T., Stehouwer C.D.A., Webers C.A.B. (2015). New ophthalmologic imaging techniques for detection and monitoring of neurodegenerative changes in diabetes: A systematic review. Lancet Diabetes Endocrinol..

[17] Sohn E.H., Van Dijk H.W., Jiao C., Kok P.H.B., Jeong W., Demirkaya N., Garmager A., Wit F., Kucukevcilioglu M., Van Velthoven M.E.J. (2016). Retinal neurodegeneration may precede microvascular changes characteristic of diabetic retinopathy in diabetes mellitus. Proc. Natl. Acad. Sci. USA.

[18] De Clerck E.E.B., Schouten J.S.A.G., Berendschot T.T.J.M., Goezinne F., Dagnelie P.C., Schaper N.C., Schram M.T., Stehouwer C.D.A., Webers C.A.B. (2018). Macular thinning in prediabetes or type 2 diabetes without diabetic retinopathy: The maastricht study. Acta Ophthalmol..

[19] Lynch S.K., Abràmoff M.D. (2017). Diabetic retinopathy is a neurodegenerative disorder. Vis. Res..

[20] Vujosevic S., Micera A., Bini S., Berton M., Esposito G., Midena E. (2016). Proteome analysis of retinal glia cells-related inflammatory cytokines in the aqueous humour of diabetic patients. Acta Ophthalmol..

[21] Vujosevic S., Micera A., Bini S., Berton M., Esposito G., Midena E. (2015). Aqueous humor biomarkers of müller cell activation in diabetic eyes. Investig. Ophthalmol. Vis. Sci..

[22] Battelino T., Danne T., Bergenstal R.M., Amiel S.A., Beck R., Biester T., Bosi E., Buckingham B.A., Cefalu W.T., Close K.L. (2019). Clinical targets for continuous glucose monitoring data interpretation: Recommendations from the international consensus on time in range. Diabetes Care.

[23] World Health Organization (2011). Use of Glycated Haemoglobin (HbA1c) in Diagnosis of Diabetes Mellitus.

[24] Frizziero L., Midena G., Longhin E., Berton M., Torresin T., Parrozzani R., Pilotto E. (2020). Early retinal changes by OCT angiography and multifocal electroretinography in diabetes. J. Clin. Med..

[25] Pilotto E., Nacci E.B., Ferrara A.M., De Mojà G., Zovato S., Midena E. (2020). Macular perfusion impairment in von Hippel-Lindau disease suggests a generalized retinal vessel alteration. J. Clin. Med..

[26] Pilotto E., Nacci E.B., De Mojà G., Ferrara A.M., Parrozzani R., Londei D., Zovato S., Midena E. (2021). Structural and microvascular changes of the peripapillary retinal nerve fiber layer in von Hippel–Lindau disease: An OCT and OCT angiography study. Sci. Rep..

[27] Midena E., Torresin T., Longhin E., Midena G., Pilotto E., Frizziero L. (2021). Early microvascular and oscillatory potentials changes in human diabetic retina: Amacrine cells and the intraretinal neurovascular crosstalk. J. Clin. Med..

[28] Gołębiewska J., Olechowski A., Wysocka-Mincewicz M., Odrobina D., Baszyńska-Wilk M., Groszek A., Szalecki M., Hautz W. (2017). Optical coherence tomography angiography vessel density in children with type 1 diabetes. PLoS ONE.

[29] Ong J.X., Fawzi A.A. (2022). Perspectives on diabetic retinopathy from advanced retinal vascular imaging. Eye.

[30] Rosen R.B., Andrade Romo J.S., Krawitz B.D., Mo S., Fawzi A.A., Linderman R.E., Carroll J., Pinhas A., Chui T.Y.P. (2019). Earliest evidence of preclinical diabetic retinopathy revealed using optical coherence tomography angiography perfused capillary density. Am. J. Ophthalmol..

[31] El-Fayoumi D., Badr Eldine N.M., Esmael A.F., Ghalwash D., Soliman H.M. (2016). Retinal nerve fiber layer and ganglion cell complex thicknesses are reduced in children with type 1 diabetes with no evidence of vascular retinopathy. Investig. Ophthalmol. Vis. Sci..

[32] Gołȩbiewska J., Olechowski A., Wysocka-Mincewicz M., Baszyńska-Wilk M., Groszek A., Czeszyk-Piotrowicz A., Szalecki M., Hautz W. (2018). Choroidal thickness and ganglion cell complex in pubescent children with type 1 diabetes without diabetic retinopathy analyzed by spectral domain optical coherence tomography. J. Diabetes Res..

[33] Prada D., Harris A., Guidoboni G., Siesky B., Huang A.M., Arciero J. (2016). Autoregulation and neurovascular coupling in the optic nerve head. Surv. Ophthalmol..

[34] Simó R., Hernández C., Porta M., Bandello F., Grauslund J., Harding S.P., Aldington S.J., Egan C., Frydkjaer-Olsen U., García-Arumí J. (2019). Effects of topically administered neuroprotective drugs in early stages of diabetic retinopathy: Results of the EUROCONDOR clinical trial. Diabetes.

[35] Xia Z., Chen H., Zheng S. (2020). Alterations of retinal pigment epithelium-photoreceptor complex in patients with type 2 diabetes mellitus without diabetic retinopathy: A cross-sectional study. J. Diabetes Res..

[36] Énzsöly A., Szabó A., Kántor O., Dávid C., Szalay P., Szabó K., Szél Á., Németh J., Lukáts Á. (2014). Pathologic alterations of the outer retina in streptozotocin-induced diabetes. Investig. Ophthalmol. Vis. Sci..

[37] Jonsson K.B., Frydkjaer-Olsen U., Grauslund J. (2016). Vascular changes and neurodegeneration in the early stages of diabetic retinopathy: Which comes first?. Ophthalmic Res..

[38] Hegazy A.I., Zedan R.H., Macky T.A., Esmat S.M. (2017). Retinal ganglion cell complex changes using spectral domain optical coherence tomography in diabetic patients without retinopathy. Int. J. Ophthalmol..

[39] Kern T.S., Barber A.J. (2008). Retinal ganglion cells in diabetes. J. Physiol..

[40] Dehghani C., Srinivasan S., Edwards K., Pritchard N., Russell A.W., Malik R.A., Efron N. (2017). Presence of peripheral neuropathy is associated with progressive thinning of retinal nerve fiber layer in type 1 diabetes. Investig. Ophthalmol. Vis. Sci..

[41] Kern T.S., Berkowitz B.A. (2015). Photoreceptors in diabetic retinopathy. J. Diabetes Investig..

[42] Wysocka-Mincewicz M., Baszyńska-Wilk M., Gołębiewska J., Olechowski A., Byczyńska A., Hautz W., Szalecki M. (2020). Influence of metabolic parameters and treatment method on OCT angiography results in children with type 1 diabetes. J. Diabetes Res..

[43] de Carlo T.E., Chin A.T., Bonini Filho M.A., Adhi M., Branchini L., Salz D.A., Baumal C.R., Crawford C., Reichel E., Witkin A.J. (2015). Detection of microvascular changes in eyes of patients with diabetes but not clinical diabetic retinopathy using optical coherence tomography angiography. Retina.

[44] Sacconi R., Casaluci M., Borrelli E., Mulinacci G., Lamanna F., Gelormini F., Carnevali A., Querques L., Zerbini G., Bandello F. (2019). Multimodal imaging assessment of vascular and neurodegenerative retinal alterations in type 1 diabetic patients without fundoscopic signs of diabetic retinopathy. J. Clin. Med..

