# Supplementary material for: Retinal Microvascular and Neuronal Changes Are Also Present, Even If Differently, in Adolescents with Type 1 Diabetes without Clinical Diabetic Retinopathy
Source: J Clin Med. 2022 Jul 8;11(14):3982. doi: 10.3390/jcm11143982 (PMC9323684; doi:10.3390/jcm11143982)
Supplement: Supplementary file 1 [file jcm-11-03982-s001.zip › jcm-1765204-supplementary.pdf]

Table S1. Full retina and single retinal layers thickness

	HC mean±SD (mm)	noDR mean±SD (mm)	DR mean±SD (mm)	p-value noDR vs HC	p-value DR vs HC	p-value DR vs noDR
**Full retina thickness**						
C	271.0±14.7	279.9±20.2	271.8±17.3	**0.0268**	0.8474	0.8488
IN	347.8±13.4	353.8±16.2	342.3±13.2	0.3012	0.2219	0.2596
IS	349.4±13.1	352.8±15.7	341.4±10.8	0.2306	0.2711	0.2644
IT	333.0±13.3	337.5±16.2	329.0±11.8	0.2775	0.5113	0.7661
II	343.8±13.3	348.5±19.9	341.2±11.5	0.2568	0.6852	0.9600
EN	324.1±14.3	328.7±13.4	321.6±10.8	0.2774	0.6885	0.9124
ES	304.5±13.0	312.5±12.6	303.4±9.4	**0.0455**	0.8885	0.6305
ET	287.8±12.2	296.3±12.8	292.2±12.7	**0.0361**	0.4014	0.3355
EI	294.2±12.1	302.3±11.8	295.2±11.4	**0.0460**	0.8193	0.9131
**Macular Retinal Nerve Fibers Layer thickness**						
C	11.9±1.5	12.7±1.6	12.7±1.5	0.4005	0.4989	0.2170
IN	20.5±1.4	21.4±2.6	19.8±2.3	0.2625	0.4957	0.7408
IS	24.2±2.5	24.3±2.9	22.5±2.4	0.9873	0.1067	0.6342
IT	16.0±0.8	16.5±1.1	16.2±1.2	0.5987	0.8718	0.3414
II	24.9±2.2	26.2±3.2	23.9±2.9	0.1169	0.3133	0.3259
EN	49.4±5. 3	50.5±6.0	46.5±3.5	0.1662	0.0066	0.0141
ES	37.1±4.3	38.6±3.7	35.8±3.1	0.0805	0.1978	0.1671
ET	17.6±0.9	18.0±1.2	17.5±1.4	0.6461	0.8862	0.4862
EI	39.8±3.2	42.3±5.5	39.4±3.1	**0.0022**	0.6546	0.1364
**Inner Plexiform Layer thickness**						
C	20.9±2.7	22.7±4.1	20.7±3.3	**0.0111**	0.8305	0.4228
IN	45.2±2.4	45.6±3.2	42.5±2.4	0.6973	**0.0093**	0.0631
IS	43.6±2.2	44.2±2.9	41.6±2.6	0.4454	0.0610	0.1291
IT	43.5±2.7	44.0±3.4	40.9±2.4	0.5491	0.0538	0.0632
II	43.4±2.3	44.3±3.0	42.6±2.1	0.2487	0.4081	0.6897
EN	31.1±3.5	31.4±2.3	31.2±1.9	0.7098	0.9449	0.1512
ES	29.7±2.5	31.0±2.0	29.7±1.4	0.1069	0.9234	0.8690
ET	32.8±2.4	34.2±2.6	33.9±3.0	0.0599	0.3115	0.1795
EI	28.8±2.8	29.7±2.3	29.0±1.6	0.2745	0.9261	0.3884
**Inner Nuclear Layer thickness**						
C	17.3±3.2	19.3±5.7	17.8±3.2	**0.0113**	0.5752	0.4391
IN	40.3±4.5	40.4±3.7	38.5±2.1	0.9759	0.1953	0.2561
IS	40.4±2.8	41.1±3.1	40.5±1.8	0.4230	0.8378	0.7676
IT	37.7±2.7	37.8±3.5	37.2±2.8	0.8471	0.8333	0.7982
II	40.1±4.0	40.2±3.2	38.0±2.3	0.9076	0.1411	0.1468
EN	36.6±4.2	36.1±2.2	34.8±1.9	0.5101	0.2022	0.6208
ES	32.8±2.7	33.4±2.3	32.5±1.4	0.4499	0.9268	0.9309
ET	35.1±2.0	35.9±2.2	34.5±1.2	0.3683	0.7273	0.5595
EI	32.4±3.3	32.6±2.2	31.7±1.7	0.8285	0.6688	0.9388
**Outer Plexiform Layer thickness**						
C	24.7±5.3	25.7±4.1	26.6±3.0	0.3102	0.3503	0.5631
IN	33.5±9.1	32.1±5.4	31.2±3.2	0.1829	0.2640	0.5088
IS	35.2±9.2	35.6±7.8	39.9±9.0	0.6491	**0.0228**	**0.0063**
IT	30.5±2.5	30.6±2.9	31.3±2.6	0.9119	0.7004	0.6528
II	32.8±7.7	30.7±4.5	30.7±2.5	**0.0422**	0.2965	0.9700
EN	28.1±2.9	27.5±2.3	27.3±2.2	0.6288	0.7001	0.8201
ES	26.6±3.0	27.1±3.3	27.4±3.5	0.6012	0.7004	0.8838
ET	26.8±1.8	26.8±1.5	27.0±1.4	0.9892	0.9466	0.9351

EI	26.1±2.8	25.9±1.6	25.3±1.4	0.8737	0.7001	0.6409
**Outer Nuclear Layer thickness**						
C	88.1±8.9	87.5±9.3	84.7±9.6	0.7531	0.4280	0.8705
IN	71.2±10.8	72.2±8.9	71.4±10.8	0.4670	0.7994	0.5442
IS	68.4±12.1	67.6±9.9	60.1±9.1	0.6771	**0.0269**	**0.0139**
IT	72.0±8.1	72.8±7.4	70.2±7.7	0.7294	0.7322	0.9556
II	67.4±8.5	70.0±8.4	68.0±9.5	0.2106	0.7165	0.8053
EN	59.5±6.8	60.9±6.6	59.4±6.3	0.4971	0.8618	0.6063
ES	62.9±6.6	63.5±6.3	60.0±6.1	0.7854	0.5080	0.5826
ET	59.8±5.7	61.3±5.7	59.3±6.3	0.4586	0.9539	0.8088
EI	55.6±5.7	56.6±6.3	54.2±7.8	0.6223	0.8383	0.9710

HC: healthy controls; noDR: eyes without diabetic retinopathy; DR: eyes with diabetic retinopathy; SD: standard deviation. Sectors of ETDRS grid: C: central, IN: internal nasal, IS: internal superior, IT: internal temporal, II: internal inferior, EN: external nasal, ES: external superior, ET: external temporal, EI: external inferior. Significant p-value in bold.
